# Comprehensive quantitative determination of aquifer confinement based on tidal response of well water level and its application in North China

**DOI:** 10.1038/s41598-024-59909-4

**Published:** 2024-04-24

**Authors:** Chenyue Hu, Xin Liao, Yun Shi, Chunguo Liu, Rui Yan, Xiaoyang Lian, Zhenyu Wang, Luming Zhang

**Affiliations:** 1 School of Ecology and Environment, Institute of Disaster Prevention, Beijing, 101601 China; 2 Hebei Key Laboratory of Resource and Environmental Disaster Mechanism and Risk Monitoring, Sanhe, 065201 China; 3 Affiliation State Key Laboratory of Earthquake Dynamics, Institute of Geology, China Earthquake Administration, Beijing, 100029 China; 4 China Earthquake Network Center, Beijing, 100045 China; 5 Institute of Geophysics, China Earthquake Administration, Beijing, 100081 China

**Keywords:** Hydrology, Hydrology

## Abstract

Aquifer confinement represents a pivotal property that significantly influences the vulnerability and contamination risk of groundwater resources. Several methods have been proposed for determining aquifer confinement by analyzing the response of well water level to Earth tides and atmospheric tides. In this study, we evaluated the performance of the existing single methods and put forward an optimized comprehensive approach. We compared the determination results of the three single methods with those of a comprehensive method using water-level data from 39 earthquake precursor monitoring wells in North China. The results demonstrate that the comprehensive method effectively determined aquifer confinement, significantly reducing the uncertainty associated with the three single methods. The application of the comprehensive method in North China reveals that aquifer confinement may undergo temporal variations during long-term continuous observation, especially in areas where the confining properties of aquifers may vary due to human activities and earthquakes. In such areas, the comprehensive method facilitates accurate assessment of groundwater vulnerability, as well as the potential dispersion of underground pollutants.

## Introduction

Confined aquifers are isolated from surface conditions and are thus better protected from pumping or contamination from shallower zones^1^. Aquifer confinement is closely related to the safety of the groundwater environment. In some countries, a large amount of wastewater is generated during oil/gas exploitation^2–5^ and industrial production and is discharged into deep aquifers covered by thick aquitards^6–8^. This practice aims to protect shallow groundwater and potable water sources from injected wastewater, considering the vulnerability of the aquifer. However, such practices implicitly assume that deep aquifers, which are covered by thick low-permeability rocks, are confined. This means that there is no apparent hydraulic connection between the deep (confined) aquifer and the shallow aquifer, and it is expected that the injected fluids will not migrate to the shallow groundwater^2^. Given the role of confinement in assessing aquifer vulnerability and protecting water resource security, there is an urgent demand for an effective method to accurately determine the confinement of aquifers.

Many factors can affect aquifer confinement. For example, earthquakes may cause aquifers to change from a confined to a semi-confined type^9–13^, and rainfall recharge may also affect the confinement of shallow aquifers^14,15^. Identifying changes in aquifer confinement is important for monitoring changes in the vertical hydraulic connection between aquifers, which may affect their vulnerability^16^, the safety of the groundwater supply^2,17^, and the security of underground wastewater storage. The most commonly used traditional methods for determining aquifer confinement is based on the well log at the time of well formation are qualitative and only representative of a certain time while ignoring the dynamic temporal changes in confinement^17–19^. Therefore, it is necessary to develop a quantitative method for enables effective determination of aquifer confinement.

Several quantitative methods have recently been proposed for determining aquifer confinement based on the tidal response of well water level (named “well tide”)^2,9,18,20–22^. An increasing number of studies are using tidal methods to investigate the effects of human activities and earthquakes on aquifer confinement^23,24^. However, the effectiveness and stability of existing tidal determination methods have not been extensively assessed. In this study, we conducted a rigorous assessment of a proposed tidal determination method and observed certain levels of uncertainty and instability in the existing approaches (see Section “Application in North China”). To address this issue, we proposed a comprehensive method that integrated multiple determination criteria from different methods to enhance the accuracy of aquifer confinement determination. To evaluate the performance of our comprehensive method, we utilized long-term water-level data obtained from the continuous monitoring of 39 earthquake precursor monitoring wells in North China. This extensive dataset serves as practical validation for the effectiveness of our comprehensive method.

## Observation

**Figure 1:**
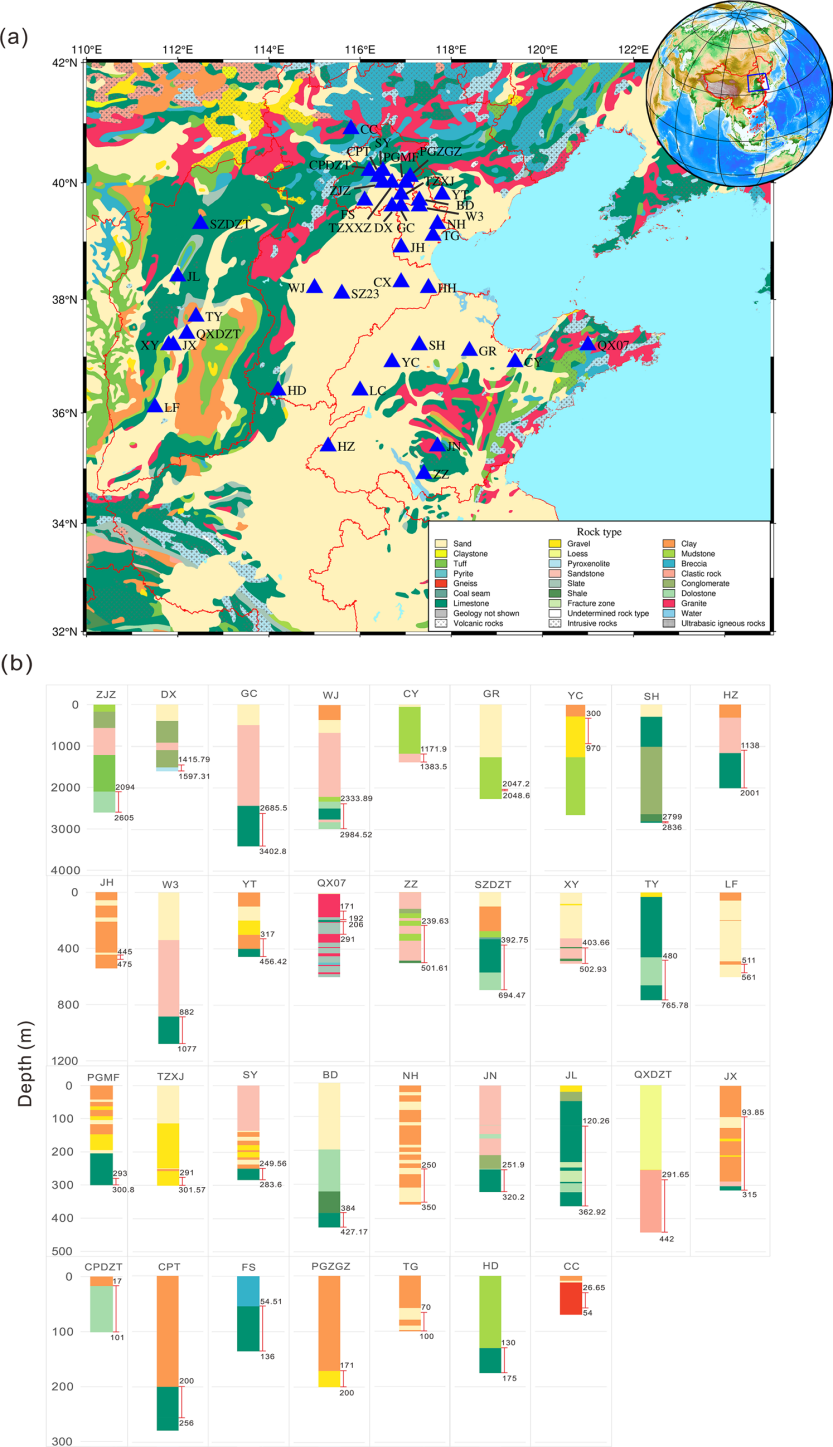
Well distribution and logs (with aquifer depth) in North China. (**a**) Well distribution. Blue triangles represent the wells (Table S1). The hydrogeological map was modified from Steinshouer et al.^28^. (**b**) Logs of wells. The red lines and corresponding numbers denote the depth of the observation aquifer (the screened section), measured in meters. The well logs were obtained from the Seismic Monitoring Records of China (2002–2007)^26^.

This study focused on the administratively distinct regions of Beijing, Tianjin, Hebei, Shandong, and Shanxi municipality/province in the North China region (Fig. 1). Across a large geological regime in North China (Fig. 1), we employed the tidal and barometric responses of groundwater as proxies to systematically investigating aquifer confinement, taking into account the depth and lithology of aquifers (Table S1). The North China region is situated on a Precambrian basement, with the upper few kilometers primarily composed of relatively homogeneous Paleozoic and Mesozoic carbonate rocks^25^. The lithology of the aquifers exhibits a considerable degree of uniformity (Table S1; Fig. 1), thereby minimizing the potential influence of topographical and lithological heterogeneity on the comparison of tidal and barometric responses among the different wells^2^.

To facilitate earthquake precursor monitoring, the China Earthquake Administration has established a nationwide seismic subsurface fluid monitoring network, which has provided a large amount of long-term continuous high-frequency data that was utilized in this study (Table S1). We carefully selected 39 wells based on the catalogs of the municipality/provincial earthquake administrations (Seismic Monitoring Records of China, 2002–2007)^26^, employing the following criteria: (a) ensuring the high quality of tidal and barometric response in the well water level data, and (b) selecting wells located at least 20 km away from the coastline to minimize the influence of ocean tides^27^. The distribution and the key information of the selected wells are presented in Fig. 1a and Table S1, respectively, while the lithological logs are displayed in Fig. 1b. Among these wells, the deepest well is the GC well in Tianjin Municipality with a depth of 3402.8 m, while the shallowest well is the CC well in Hebei Province with a depth of 69.45 m.

The water levels in the 39 wells were predominantly determined using high-precision digital water-level recordings, guaranteeing accuracy up to 1 mm. These recordings spanned a time period of 10 years, providing a comprehensive dataset for analysis. Digital pressure transducers were utilized to measure water levels, with a range of 0–10 m and a sampling interval of 1 min. The relevant well parameters and observed aquifers for each of the 39 wells are provided in Table S1.

However, logs for some wells were not collected.

## Methods for determining aquifer confinement

### Methods based on tidal response

**Figure 2:**
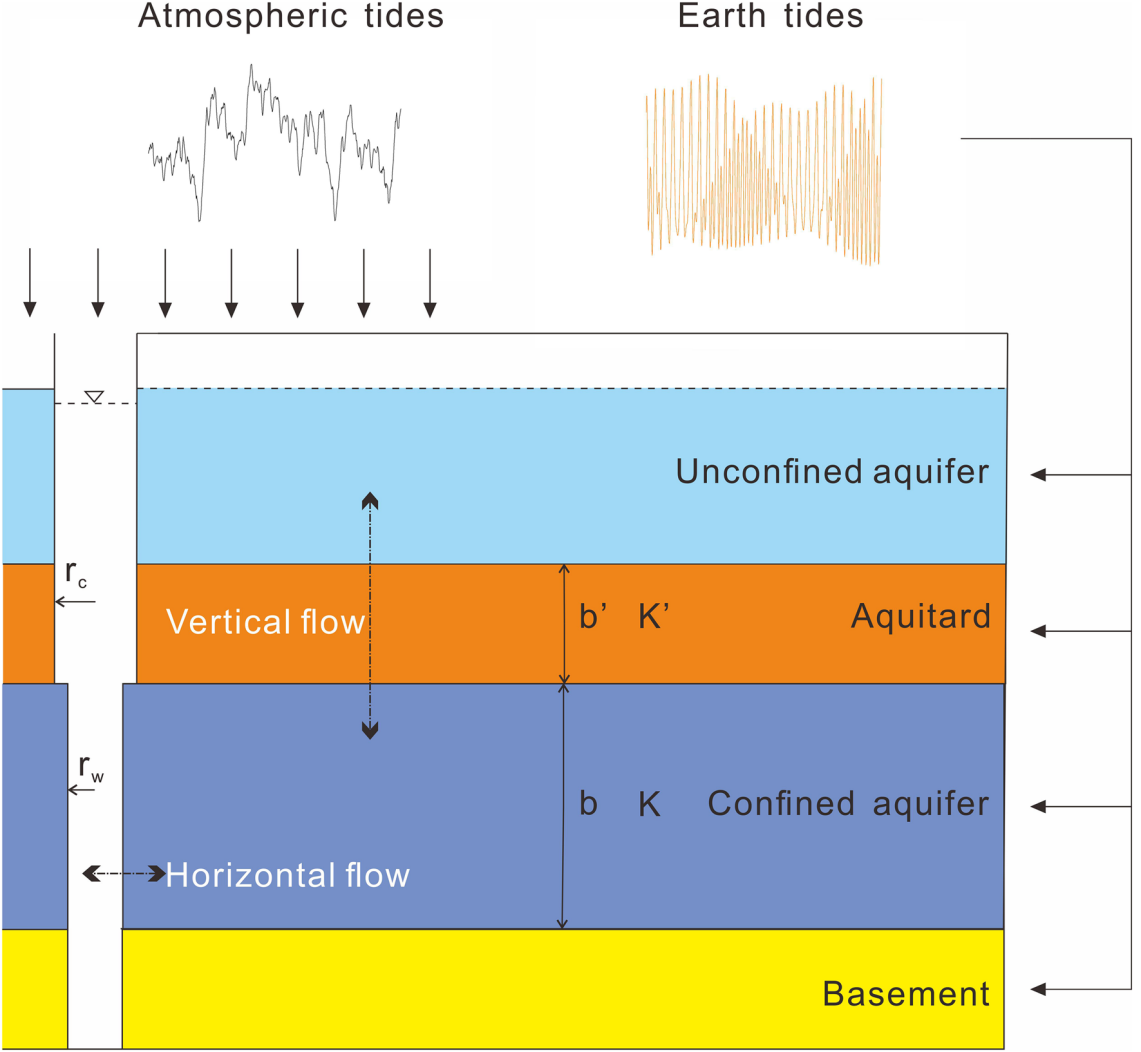
Schematic diagram of a leaky aquifer. $b{\prime}$ and $K{\prime}$ represent the equivalent thickness and vertical hydraulic conductivity of the aquitard, respectively; $b$ and *K* represent the thickness and hydraulic conductivity of the aquifer, respectively; ${r}_{w}$ (m) represents the radius of the screened portion of the well; and ${r}_{c}$ (m) represents the inner radius of the well casing in which the water level fluctuates with tides.

Gravitational forces exerted on the Earth by the motions of the Moon and Sun and centrifugal forces due to rotation act throughout the volume of the Earth’s body, producing small latitudinal and longitudinal strains within the solid crust of the Earth and leading to the horizontal and vertical groundwater flow in aquifer-aquitard systems^27,29,30^. This phenomenon is commonly known as the tidal response of well water level (Fig. 2). Herein, we summarize the three single methods employed to quantitatively determine aquifer confinement, based on the response of well water level to the Earth tides and atmospheric tides (also see Table 1).

**Table 1:** Tidal response characteristics of well water level for different aquifer confinements based on three published single methods.

	Unconfined aquifer	Semi-confined aquifer^a^	Confined aquifer	References
First method	Phase shift of the M_2_ wave is positive	Phase shift of the M_2_ wave consistently has positive and negative values	Phase shift of the M_2_ wave is negative	Shi and Wang, (2016)^9^ and Zhang et al. (2021)^2^
Second method	Amplitude and phase shift of the M_2_ wave are negatively correlated	Amplitude and phase shift of the M_2_ wave are consistently positively and negatively correlated	Amplitude and phase shift of the M_2_ wave are positively correlated	Liao and Wang (2018)^31^ and Liao et al. (2022)^14^
Third method	Amplitude of the M_2_ wave is smaller than that of the S_2_ wave	Amplitudes of the M_2_ and S_2_ waves are equivalent or alternate	Amplitude of the M_2_ wave is larger than that of the S_2_ wave	Rahi and Halihan (2013)^1^, Hussein et al. (2013)^16^, and Odling et al. (2015)^32^

^a^The criteria proposed in this study for determining semi-confined aquifers are the combination with the tidal response characteristics observed in both confined and unconfined ones.

The first method relies on the phase shift value of the M_2_ wave^9,23,31^. According to the theoretical models of tidal response to the Earth tides (Fig. S1)^16,23,33,34^, it has been observed that in confined aquifers, the phase shift is negative, whereas when in the unconfined aquifer, the phase shift is positive. Thus, the phase shift of the M_2_ wave can be used to determine aquifer confinement^9,22^.

The second method utilizes the amplitude–phase shift relationship of M_2_ waves^23,31,34^. Based on the theoretical models of tidal response to the Earth tides (Fig. S1)^23,33–35^, it has been observed that in confined aquifers, the well tide is primarily affected by transmissivity ($T$), resulting in a direct proportional relationship between the amplitude and phase shift. Conversely, in unconfined aquifers, the well tide is mainly affected by leakage ($u$), leading to an inversely proportional relationship between the amplitude and the phase shift. Therefore, the amplitude–phase shift relationship of M_2_ waves can be used to determine aquifer confinement^14,31^.

The third method relies on the disparity in amplitude between the M_2_ and S_2_ waves^1,36^. Drawing upon the theoretical models of tidal response to the Earth tides^16,23,33^ and atmospheric tides^33,37,38^, it has been observed that in unconfined aquifers, resulting in a significantly larger amplitude for the S_2_ wave compared to the M_2_ wave. Conversely, in confined aquifers, the tidal response of the water level is predominantly influenced by Earth’s tide loading, leading to a significantly smaller amplitude for the S_2_ wave compared to the M_2_ wave^1^. Therefore, the disparity in amplitude between the M_2_ and S_2_ waves can be used to determine aquifer confinement^1,16,32^.

### Comprehensive method for determining aquifer confinement

To address the potential limitations of individual methods and minimize uncertainty and instability in the results (see detailed information in Section “Reasons for differences in the determination results between methods”), we proposed a comprehensive method that combines multiple single methods. The result for the comprehensive method is obtained by summing the weighted results of the single methods, which ultimately determines the aquifer confinement (Table 2). For example, if an aquifer is determined to be confined (weighted value is 1) using two single methods, and unconfined (weighted value is − 1) using the other single method, then the comprehensive value equals 1 and the aquifer is determined to be semi-confined. If an aquifer is determined to be unconfined using two single methods, and confined using the other single method, then the comprehensive value equals − 1 and the aquifer is determined to be semi-confined.

**Table 2:** Comprehensive value for the comprehensive method, which is a summation of the weighted values from the single methods, for determining aquifer confinement.

Aquifer type	Weighted value for single methods	Comprehensive value for comprehensive method
Confined aquifer	1	2, 3
Semi-confined aquifer	0	− 1, 0, 1
Unconfined aquifer	− 1	− 3, − 2

## Application in North China

In this study, we applied three single methods and a comprehensive method based on the well tide to determine aquifer confinement in 39 wells located in North China. To analyze the water level response to Earth tides and atmospheric tides, we utilized the widely adopted software package Baytap-G^39^, with a moving window (SPAN) of 720 h and a running step (SHIFT) of 24 h. We used both the M_2_ and S_2_K_2_ tide response waves in the water-level data as constraints, as these two waves exhibited the largest amplitude and highest signal‐to‐noise ratio^34^. For practical application, we substituted the S_2_ wave with the S_2_K_2_ wave when determining aquifer confinement due to the similar frequencies of S_2_ (2 cycles/day) and K_2_ waves (2.0056 cycles/day), which cannot be distinguished using Baytap-G software. In fact, the S_2_ wave demonstrated significantly larger amplitude compared to the K_2_ wave. Furthermore, the K_2_ tidal amplitude had minimal impact on the well water level's response to atmospheric tide^34,40^. As a result, comparing the amplitudes of the M_2_ and S_2_K_2_ waves did not affect the outcomes of the third method.

The discriminant results obtained from the three single methods and the comprehensive method are summarized in Table S1. We counted the number of wells with different results and found that among the 38 wells studied, 10.53% (4 out of 38) exhibited three different results, 65.79% (25 out of 38) showed two different results, and 23.68% (9 out of 38) had consistent results. Furthermore, the difference between the results of the first and second methods was 55.26%, the difference between the results of the first and third methods was 55.26%, and the difference between the results of the second and third methods was 57.89% (Table 3). These findings, derived from field applications, indicated that the determination of aquifer confinement can yield varying results depending on the methods employed, and even the same method applied at different time periods. Evidently, when determining aquifer confinement at different time periods, a certain level of uncertainty and instability can occur when solely relying on a single method.

**Table 3:** Percentage of wells with differences between the determined results of aquifer confinement using the different methods. The smaller the ratio, the smaller the difference in the results between the different methods. We did not include the CX well in this analysis owing to changes in aquifer confinement during long-term monitoring (see Section “Reasons for differences in the determination results between methods”).

	First method (%)	Second method (%)	Third method (%)	Comprehensive method (%)
First method	–	55.26	55.26	28.95
Second method	55.26	–	57.89	31.58
Third method	55.26	57.89	–	34.21
Comprehensive method	28.95	31.58	34.21	–

We assessed whether the application of the comprehensive method could mitigate a certain level of uncertainty and enhance the stability in determining aquifer confinement based on the well tide. The disparities between the results from the comprehensive method and the first, second, and third methods were 28.95%, 31.58%, and 34.21%, respectively (Table 3). These findings indicate that the comprehensive method reduces the uncertainty associated with the three single methods from 55.26 to 28.95%, from 55.26 to 31.58%, and from 57.89 to 34.21%, respectively. Consequently, these results imply that the implementation of the comprehensive determination method can effectively diminish a certain level of uncertainty and enhance the stability of the tidal determination method.

Furthermore, a notable disparity of 50% was observed in the number of wells between the comprehensive method, which relied on the tidal response, and the traditional method based on the well log data at the time of well formation (Table S1). This observation indicates that not only do the methods themselves differ, but also the confinement status of certain aquifers changed over the monitoring period, as illustrated by well CX (see Fig. S2). Considering the potential for aquifer confinement variations during long-term continuous monitoring, particularly in areas where seismic and human activities can impact the underground structure, traditional methods may possess limitations in accurately determining aquifer confinement.

## Discussion

### Reasons for differences in the determination results between methods

The disparities in the results between the comprehensive tidal method and the single tidal methods (Table S1) can be attributed to the comprehensiveness of the determination criteria. Each method possesses distinct determination criteria and application conditions, resulting in notable variations in the determination results. The comprehensive method with more determination criteria can reduce a certain level of potential uncertainty and instability inherent in the results obtained from single tidal determination methods, while also expanding the applicability of the comprehensive method. Therefore, the comprehensive method would be more effective than a single method for determining aquifer confinement.

Further comparison of the determination results between the comprehensive tidal method and traditional methods (Table S1) reveals several underlying reasons for the observed differences. Firstly, the qualitative determination of aquifer confinement based on lithology at the time of well formation is subject to significant limitations. The results of this method typically only reflect a small portion of the surrounding area near the borehole (at the scale of meters). Particularly, in the case of heterogeneous and anisotropic subsurface formations, the effectiveness of this method is further diminished^41^.

In contrast, the comprehensive tidal method considers a larger area (around 100 m) surrounding the borehole^17,42^, providing a more comprehensive assessment. Secondly, various factors such as large earthquakes^9,20,24,43^, hydrological processes such as rainfall^14,15,31,44^, and even land subsidence (e.g., CX well; see Fig. S2)^45^ can affect aquifers confinement during long-term continuous monitoring. These factors can lead to the generation of hydraulic connections with the overlying aquifer, thereby altering the aquifer's confinement status to a semi-confined or even unconfined type.

### Analysis of factors influencing aquifer confinement

Generally, the deeper the well, the more likely the associated aquifer is to be confined. Thus, aquifer confinement is superficially affected by well depth. However, it is important to note that a greater well depth does not necessarily correspond to a thicker overlying aquitard. For example, in the case of the YC well (with a depth of 2657 m), the aquifer observed had a buried depth of only 300 m, resulting in its comprehensive determination as semi-confined. Generally, The degree of aquifer confinement is primarily influenced by the coefficient of leakage (*u* = *K*′/*b*′), which is affected by both the thickness (*b*′) and vertical hydraulic conductivity (*K*′) of the aquitard. In other words, the observed burial depth of the aquifer and the lithology of the overlying aquitard play crucial roles in determining aquitard thickness and vertical hydraulic conductivity, respectively, ultimately affecting the level of aquifer confinement.

**Figure 3:**
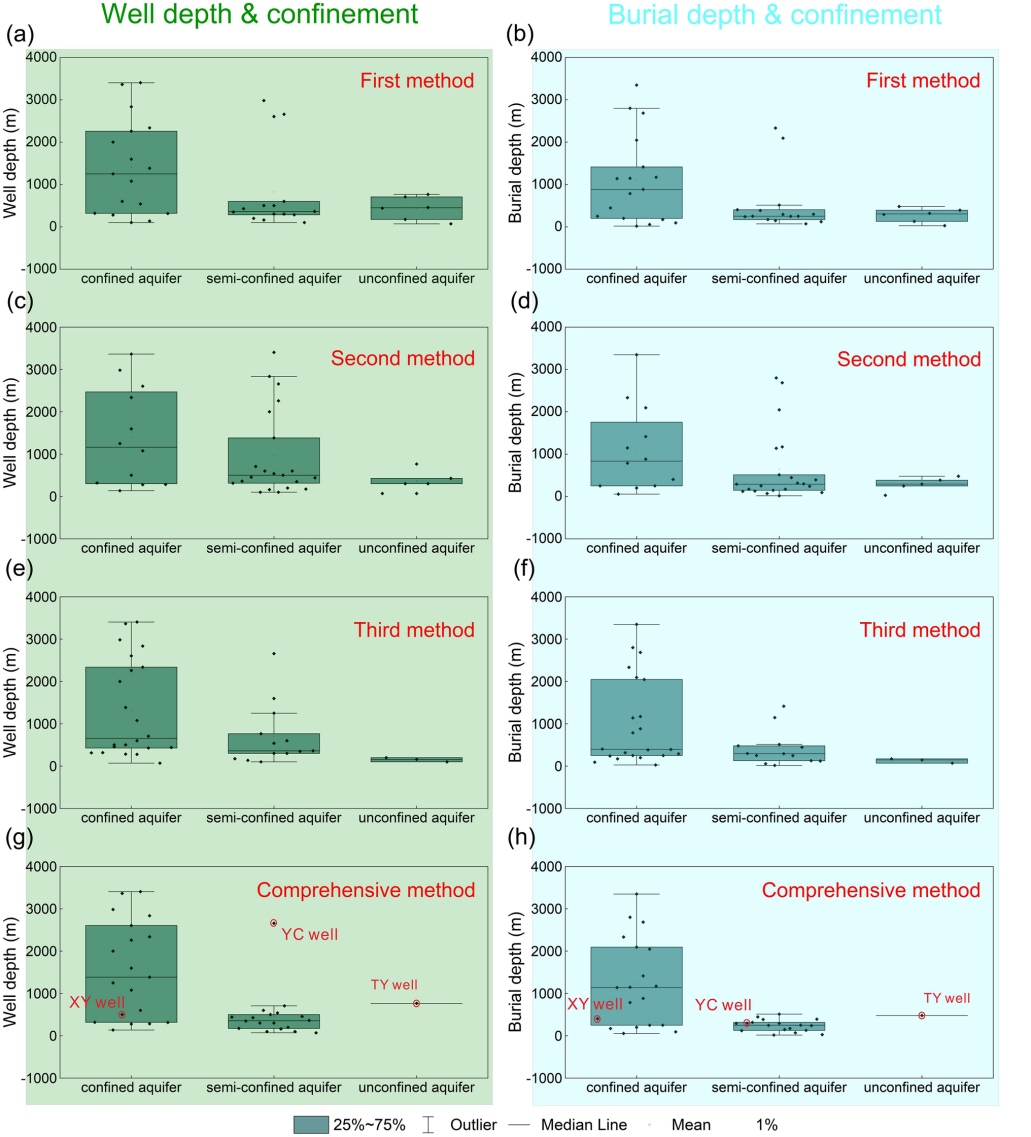
Box plots of aquifer confinement determined using different methods, including the first, second, third, and comprehensive tidal methods. Results for the (**a**, **b**) first, (**c**, **d**) second, (**e**, **f**) third, and (**g**, **h**) comprehensive methods. The graphs with dark green and light blue backgrounds indicate aquifer confinement at different well and burial depths, respectively. The graph can be used to judge the effectiveness of the tidal determination methods and analyze the relationship between aquifer confinement and well depth or burial depth.

Generally, the greater the thickness of the overlying aquitard, the less hydraulic connections are established between deep and shallow aquifers. The average burial depth for confined aquifers was significantly greater compared to semi-confined and unconfined aquifers (Fig. 3), suggesting that greater depth corresponds to stronger aquifer confinement. The findings indicate that the observed burial depth of the aquifer has a more significant influence on aquifer confinement than the lithology of the overlying aquitard, even though the lithology of overlying aquitards may vary greatly. In our study area, the deeper the burial depth of the aquifer, the more likely it is that the overlying aquitard will have weakly permeable lithology, such as shale, indicating a confined aquifer condition. This findings indicates the primary role of the observed burial depth of the aquifer in determining aquifer confinement in our study area, rather than the lithology of the overlying aquitard.

Notably, the lithology of the overlying aquitard becomes a crucial factor affecting aquifer confinement when the burial depth remains consistent. For example, although the small discrepancy in burial depth between aquifers observed in the TY and XY wells (TY: 480 m, XY: 403.66 m), the TY-well aquifer was unconfined, whereas the XY-well aquifer was confined (Fig. 3). This discrepancy in confinement can be attributed to the influence of lithology. The aquitards overlying the TY- and XY-well observed aquifers comprised relatively permeable limestone and low-permeability clay layers, respectively. Therefore, the contrasting lithologies played a role in the variation of aquifer confinement between the two wells, despite both possessing equally thick overlying aquitards.

## Conclusion

In this study, we critically examined the effectiveness of current individual tidal determination methods and subsequently proposed an enhanced, comprehensive determination method. Utilizing water level data sourced from 39 earthquake precursor monitoring wells in North China, we compared the conclusions determined by three independent single methods against those determined by the comprehensive method. The results indicated that the comprehensive method could significantly reduce the uncertainty associated with the three single methods, from 55.26 to 28.95%, from 55.26 to 31.58%, and from 57.89 to 34.21%, respectively. Furthermore, it has been observed that aquifer confinement may change during long-term continuous monitoring, especially in areas where the confining properties of aquifers are susceptible to changes caused by human activities and earthquakes. In these areas, the comprehensive method greatly contributes in the precise assessment of groundwater vulnerability and the potential dispersion of pollutants. Overall, this study provides valuable insights for assessing and understanding aquifer confinement, enhancing our ability to manage groundwater resources in dynamic environments.

### Supplementary Information

Supplementary Information 1.

Supplementary Table S1.

## Data Availability

The well-water-level data analyzed in this study can be downloaded via direct application at the China Earthquake Networks Center, National Earthquake Data Center (http://data.earthquake.cn/gcywfl/index.html; no English translation is available for the data).
