# Supplementary material for: Comprehensive quantitative determination of aquifer confinement based on tidal response of well water level and its application in North China
Source: Sci Rep. 2024 Apr 24;14:9464. doi: 10.1038/s41598-024-59909-4 (PMC11043444; doi:10.1038/s41598-024-59909-4)
Supplement: Supplementary file 1 — Supplementary Information 1. [file 41598_2024_59909_MOESM1_ESM.docx]

Supporting Information for

**Comprehensive Quantitative Determination of Aquifer Confinement Based on Tidal Response of Well Water Level and its Application in North China**

**Chenyue Hu^1, 2^, Xin Liao^1, 2, *^, Yun Shi^1, 2, 3, *^, Chunguo Liu^4^, Rui Yan^4^, Xiaoyang Lian^1, 2^, Zhenyu Wang^5^, & Luming Zhang^1, 2^**

1. School of Ecology and Environment, Institute of Disaster Prevention, Beijing 101601, China
2. Hebei Key Laboratory of Resource and Environmental Disaster Mechanism and Risk Monitoring, Sanhe 065201, China
3. State Key Laboratory of Earthquake Dynamics, Institute of Geology, China Earthquake Administration, Beijing 100029, China
4. Ch ina Earthquake Network Center, Beijing 100045, China
5. Institute of Geophysics, China Earthquake Administration, Beijing 100081, China

*Corresponding Author:

Xin Liao ([liaoxin19851224@126.com](mailto:*liaoxin19851224@126.com)); Yun Shi ([444739691@qq.com](mailto:444739691@qq.com))

**Contents of this file**

Text S1. Three methods for determining aquifer confinement

Figure S1. Theoretical curves for confined and unconfined aquifers according to Hsieh et al.’s (1987) and Doan et al.’s models (2006)

Figure S2. Determining of aquifer confinement observed in the CX well according to the comprehensive tidal determination method

**Additional Supporting Information (Files uploaded separately)**

Table S1. Basic information on wells and results of aquifer confinement in North China

**Introduction**

This Supporting Information contains the following three sections: a supporting text, two supporting figures and a supporting table.

Text S1. Three methods for determining aquifer confinement

In this study, we summarize the three single methods used to quantitatively determine aquifer confinement based on the response of the water level to the Earth and atmospheric tides. The first method is based on the phase-shift value of the M_2_ wave^1-4^. The phase shift refers to the difference between the phase of the tidal components in the well water level data and that in the theoretical volumetric tide (phase positive or negative). According to the theoretical curve of Hsieh et al.'s model (1987) (**Figure S1b, S1d**), when the leakage ($u$) is very small (<10^-9^ s^-1^), the aquifer behaves as a confined aquifer, with a negative phase shift. This is because the groundwater in the aquifer always requires time for a poroelastic response to tidal forces and to flow into or out of the well^5^. When the leakage ($u$*)* is very large (>10^-9^ s^-1^), the aquifer behaves as an unconfined aquifer, with a positive phase shift.

The second method is based on the amplitude–phase shift relationship of M_2_ waves^1-3,6^ based on the theoretical curves of Hsieh et al.’s model (1987) (**Figure S1a, S1b**) and for an unconfined aquifer (**Figure S1c, S1d**^3^) at different leakages ($u$). **Figure S1**a and b show that, for confined aquifers, an increase in $T$ causes a proportional increase in the amplitude ($A$) and phase shift ($\eta$). On the other hand, **Figure S1**c and d show that, for unconfined aquifers, an increase in *u*causes an inversely proportional decrease in the amplitude ($A$) and increase in the phase shift ($\eta$).

The third method is based on the difference in the amplitude between the M_2_ and S_2_ waves^7,8^. The M_2_ tide is influenced by the Earth's tides, thus providing a consistent Earth tidal signature, while the S_2_ signal has the same frequency as that of the daily changes in atmospheric pressure, thus providing an atmospheric signature. These two signals can be used to evaluate aquifer confinement by measuring the respective effects of the Earth and atmospheric tides. When the tidal response of the water level is mainly affected by atmospheric loading, the amplitude of the S_2_ wave is significantly larger than that of the M_2_ wave, and the aquifer is determined to be unconfined. Correspondingly, when the tidal response of the water level is mainly affected by Earth's tide loading, the amplitude of the S_2_ wave is significantly smaller than that of the M_2_ wave, and the aquifer is determined to be confined^7^.


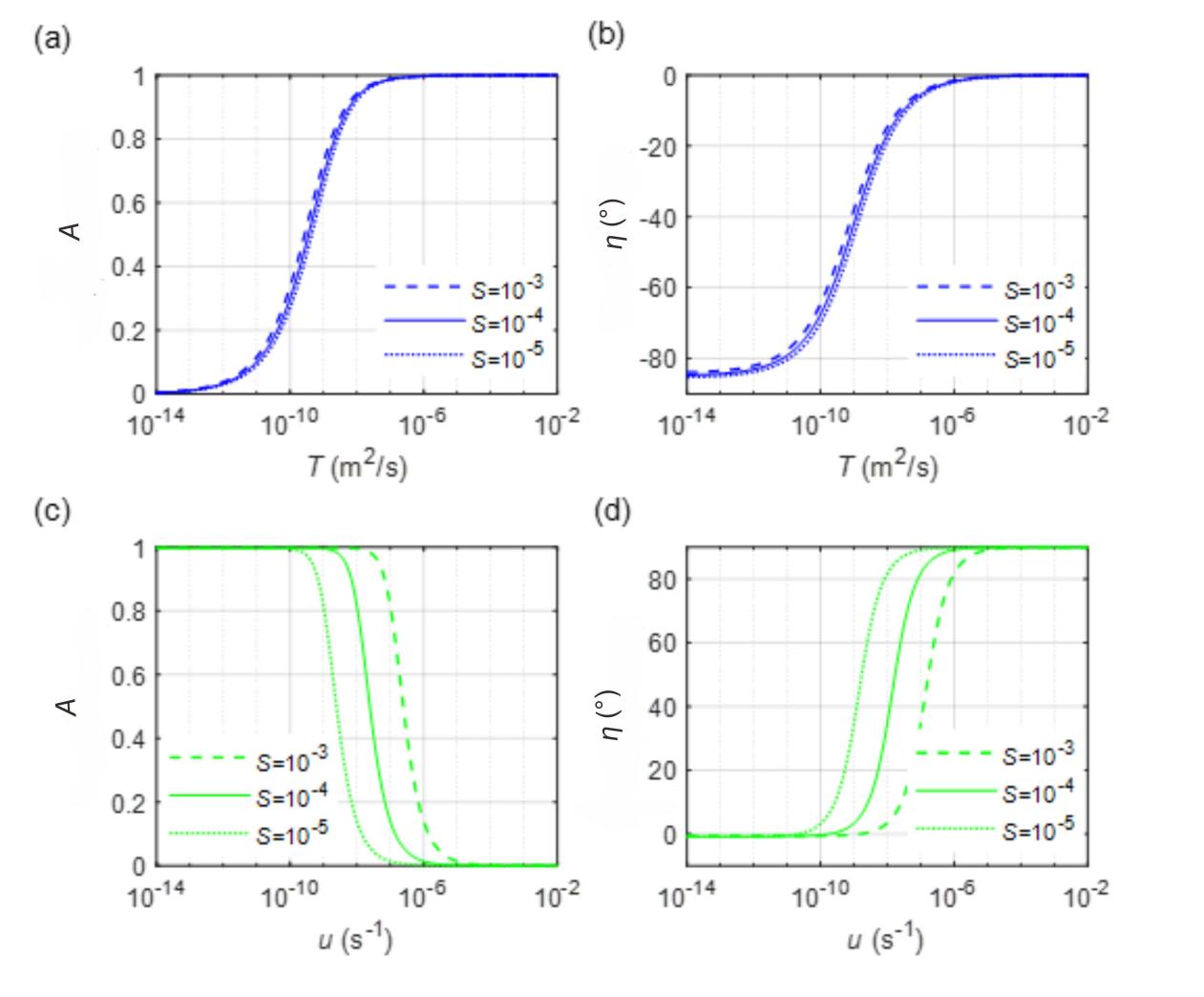


Figure S1. (a) and (b) Model prediction of amplitude ($\boldsymbol{A}$) and phase shift ($\boldsymbol{\eta}$), respectively, for the tidal response of water level to the M_2_ tide in a confined aquifer plotted against the transmissivity ($\boldsymbol{T}$) for different storativity ($\boldsymbol{S}$) (Modified from Liao et al., 2022). It shows that, between $\boldsymbol{T}$ = 10^-10^ and 10^-7^ m^2^/s, the amplitude ($\boldsymbol{A}$) and phase shift ($\boldsymbol{\eta}$) are positively correlated, with a negative phase shift. (c) and (d) Model prediction of amplitude ($\boldsymbol{A}$) and phase shift ($\boldsymbol{\eta}$), respectively, for the response of water level to the M_2_ tide in an unconfined aquifer, plotted against leakage ($\boldsymbol{u}$) for different storativity ($\boldsymbol{S}$), with $\boldsymbol{T}$ = 10^-4^ m^2^/s and r_w_ = r_c_ = 5 cm, showing that between $\boldsymbol{u}$ = 10^-9^ and 10^-7^ s^-1^, the amplitude ($\boldsymbol{A}$) and phase shift ($\boldsymbol{\eta}$) are negatively correlated, with a positive phase shift.


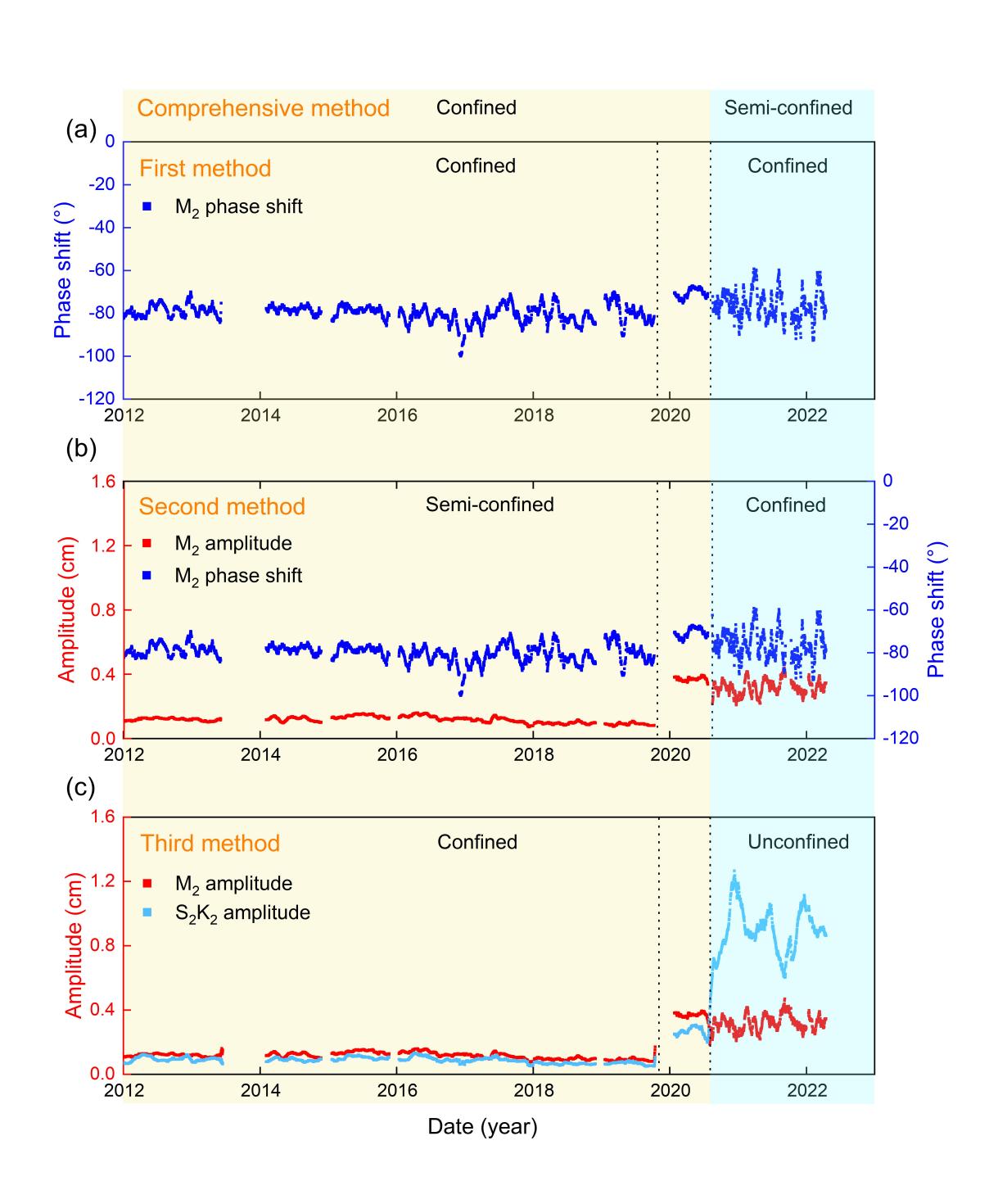


**Figure S2.** Temporal variation in the phase shift and amplitude used to determine aquifer confinement. (a), (b), and (c) show the phase shift of the M_2_ wave, amplitude and phase shift of the M_2_ tide for the CX well from 2012 to 2022, and amplitude of the tidal waves, including M_2_ and S_2_K_2_ waves, over time. The results of the comprehensive method indicate that the confinement of the aquifer changes during long-term continuous monitoring. No seismic and rainfall effects were found in either of the step-like changes in aquifer confinement. Considering that well CX is located in the land subsidence center of North China^9^, the step-like change in aquifer confinement can be attributed to non-uniform consolidation inducing failure of shallow strata, which usually act as an aquiclude.

Table S1. Basic information on wells and results of aquifer confinement in North China.

**References**

1. Wang, C.Y., Doan, M.L., Xue, L. & Barbour, A.J. Tidal response of groundwater in a leaky aquifer–Application to Oklahoma. *Water Resour. Res.* **54**(10), 8019–8033. <https://doi.org/10.1029/2018WR022793> (2018).
2. Hsieh, P.A., Bredehoeft, J.D. & Farr, J.M. Determination of aquifer transmissivity from Earth tide analysis. *Water Resour. Res.* **23**(10), 1824–1832. <https://doi.org/10.1029/WR023i010p01824> (1987).
3. Doan, M.L., Brodsky, E.E., Prioul, R. & Signer, C. Tidal Analysis of Borehole Pressure: A Tutorial, University of California, p.1-61. (2006).
4. Shi, Z. & Wang, G. Aquifers switched from confined to semiconfined by earthquakes. *Geophys. Res. Lett.* **43**(21), 166–111, 172. <https://doi.org/10.1002/2016GL070937> (2016).
5. Gao, X., Sato, K. & Horne, R.N. General solution for tidal behavior in confined and semiconfined aquifers considering skin and wellbore storage effects. *Water Resour. Res.* **56**(6), e2020WR027195. <https://doi.org/10.1029/2020WR027195> (2020).
6. Liao, X. & Wang, C.Y. Seasonal permeability change of the shallow crust inferred from deep well monitoring. *Geophys. Res. Lett.* **45**(20), 130–111, 136. <https://doi.org/10.1029/2018GL080161> (2018).
7. Rahi, K.A. & Halihan, T. Identifying aquifer type in fractured rock aquifers using harmonic analysis. *Ground Water.* **51**(1), 76–82. <https://doi.org/10.1111/j.1745-6584.2012.00925.x> (2013).
8. Rahi, K.A. Estimating the hydraulic parameters of the Arbuckle-Simpson aquifer by analysis of naturally induced stresses (Ph.D. dissertation). School of Geology, Oklahoma State University: Stillwater, Oklahoma. (2010).
9. Guo, H.P. et al. Land subsidence and its affecting factors in Cangzhou, North China Plain. *Front. Environ. Sci. 10*. 1053362. <https://doi.org/10.3389/fenvs.2022.1053362> (2022).
