# Supplementary material for: Comprehensive quantitative determination of aquifer confinement based on tidal response of well water level and its application in North China
Source: Sci Rep. 2024 Apr 24;14:9464. doi: 10.1038/s41598-024-59909-4 (PMC11043444; doi:10.1038/s41598-024-59909-4)
Supplement: Supplementary file 2 — Supplementary Table S1. [file 41598_2024_59909_MOESM2_ESM.docx]

**Table S1.** Basic information on wells and results of aquifer confinement in North China.

| **Serial number** | **Province/Municipality** | **Station** | **Location** | **Observation instrument** | **Well completion time** | **Record period^#^** | **Well diameter (mm)** | **Casing interval (m)** | **Screen interval (m)** | **Well depth (m)** | **Aquifer thickness (m)** | **Aquifer lithology** | **Aquitard thickness (m)** | **Aquitard lithology** | **Traditional method*** | **First method** | **Second method** | **Third method** | **Comprehensive method** |
| --- | --- | --- | --- | --- | --- | --- | --- | --- | --- | --- | --- | --- | --- | --- | --- | --- | --- | --- | --- |
| *1* | *Beijing* | *ZJZ* | 116.5°E 40°N | LN-3A | 1984 | 2007-2021 | 160 | 252 | 62 | 2605 | 521 | Dolostone & andesite | 2079 | Mudstone | Confined | Semi-confined | Confined | Confined | Confined |
| *2* |  | *PGMF* | 117°E 40°N | ZKGD3000 | 1968 | 2011-2021 | / | / | / | 300.8 | 7.8 | Limestone | 293 | Clay | Confined | Semi-confined | Unconfined | Semi-confined | Semi-confined |
| *3* |  | *CPDZT* | 116.2°E 40.2°N | LN-3 | 1984 | 2005-2013 | / | / | / | 101 | 84 | Dolostone | 17 | Clay | Confined | Confined | Semi-confined | Semi-confined | Semi-confined |
| *4* |  | *CPT* | 116.4°E 40.1°N | SWY-1 | 1980 | 2001-2021 | / | / | / | 279 | 56 | Limestone | 200 | Clay | Confined | Confined | Confined | Confined | Confined |
| *5* |  | *TZXXZ* | 116.7°E 40.0°N | SWY-1 | 1975 | 2001-2022 | / | / | / | 162 | 16 | Sandstone | 146 | / | Confined | Semi-confined | Semi-confined | Unconfined | Semi-confined |
| *6* |  | *TZXJ* | 116.9°E 39.8°N | ZKGD3000 | 1969 | 2011-2021 | / | / | / | 301.57 | 42.57 | Boulder & gravel | 249 | Clay & gravel | Confined | Semi-confined | Unconfined | Semi-confined | Semi-confined |
| *7* |  | *DX* | 116.7°E 39.6°N | LN-3A | 1980 | 2012-2015 | 120 | 1415.79 | 182 | 1597.31 | 181.52 | Conglomerate | 1415.79 | Sand | Confined | Confined | Confined | Semi-confined | Confined |
| *8* |  | *FS* | 116.1°E 39.7°N | LN-3A | 1969 | 2012-2022 | / | / | / | 136 | 81.49 | Limestone | 54.51 | Conglomerate | Confined | Confined | Confined | Semi-confined | Confined |
| *9* |  | *PGZGZ* | 117.1°E 40.1°N | SWY-1 | 1989 | 2012-2022 | / | / | / | 200 | 29 | Boulder & gravel | 171 | Clay | Confined | Semi-confined | Semi-confined | Unconfined | Semi-confined |
| *10* |  | *SY* | 116.5°E 40.2°N | SWY-1 | 1970 | 2001-2022 | / | / | / | 283.6 | 34.04 | Limestone | 249.56 | Clay | Confined | Semi-confined | Confined | Confined | Confined |
| *11* | *Tianjin* | *BD* | 117.3°E 39.7°N | LN-3 | 1970 | 2001-2015 | 350 | 197 | 230.17 | 427.17 | 217 | Limestone | 205 | Shale | Confined | Semi-confined | Unconfined | Confined | Semi-confined |
| *12* |  | *W3* | 117.3°E 39.6°N | LN-3A | 1978 | 2011-2022 | 60 | / | / | 1077 | 195 | Limestone | 882 | Mudstone | Confined | Confined | Confined | Confined | Confined |
| *13* |  | *GC* | 116.9°E 39.6°N | LN-3A | 1978 | 2001-2013 | 240 | / | / | 3402.81 | 717.3 | Limestone | 2685.5 | Mudstone & shale | Confined | Confined | Semi-confined | Confined | Confined |
| *14* |  | *TG* | 117.6°E 39.1°N | LN-3A | 1969 | 2012-2022 | / | / | / | 100 | 30 | Sand | 70 | Clay | Confined | Semi-confined | Semi-confined | Unconfined | Semi-confined |
| *15* |  | *JH* | 116.9°E 38.9°N | LN-3 | 1976 | 2012-2017 | 152 | / | / | 540.9 | 30 | Sand | 445 | Clay | Confined | Confined | Semi-confined | Semi-confined | Semi-confined |
| *16* |  | *NH* | 117.7°E 39.3°N | LN-3A | 1976 | 2012-2022 | / | / | / | 350 | 100 | Sand | 250 | Clay & sand | Confined | Semi-confined | Semi-confined | Semi-confined | Semi-confined |
| *17* | *Hebei* | *YT* | 117.8°E 39.8°N | LN-3 | 1980 | 2012-2022 | / | / | / | 456.4 | 139.42 | Limestone | 317 | Gravel & clay | Confined | Unconfined | Semi-confined | Confined | Semi-confined |
| *18* |  | *CX* | 116.9°E 38.3°N | SWY-1 | 1976 | 2012-2022 | / | / | / | 2362 | 480 | Limestone | 1390 | / | Confined | Confined | Semi-confined | Confined→Unconfined | Confined→Semi-confined |
| *19* |  | *HH* | 117.5°E 38.2°N | SWY-1 | 1971 | 2012-2022 | / | / | / | 1250 | 105 | Limestone | 1145 | / | Confined | Confined | Confined | Semi-confined | Confined |
| *20* |  | *WJ* | 115°E 38.2°N | SWY-1 | 1989 | 2012-2022 | / | / | / | 2984 | 650.63 | Limestone & dolostone | 2333.89 | Mudstone & sandstone | Confined | Semi-confined | Semi-confined | Confined | Semi-confined |
| *21* |  | *SZ23* | 115.6°E 38.1°N | SWY-1 | 1977 | 2012-2022 | / | / | / | 3364 | 17 | Limestone & dolostone | 3347 | Mudstone | Confined | Confined | Confined | Confined | Confined |
| *22* |  | *HD* | 114.2°E 36.4°N | SWY-1 | 1977 | 2012-2021 | / | / | / | 175 | 45 | Limestone | 130 | Limestone & mudstone | Confined | Unconfined | Semi-confined | Semi-confined | Semi-confined |
| *23* |  | *CC* | 115.8°E 40.9°N | LN-3 | 1977 | 2012-2018 | 146 | 69.45 | / | 69.45 | 27.35 | Granite | 26.65 | Clay | Confined | Unconfined | Unconfined | Confined | Semi-confined |
| *24* | *Shandong* | *CY* | 119.4°E 36.9°N | LN-3A | 1974 | 2012-2022 | 140 | 1171.9 | 211.6 | 1383.5 | 211.1 | Sandstone | 1171.9 | Mudstone | Confined | Confined | Semi-confined | Confined | Confined |
| *25* |  | *GR* | 118.4°E 37.1°N | LN-3A | 1971 | 2012-2022 | 140 | 2260 | 0.9 | 2260 | 1.4 | Sandstone | 2047.2 | Mudstone | Confined | Confined | Semi-confined | Confined | Confined |
| *26* |  | *YC* | 116.7°E 36.9°N | LN-3A | 1980 | 2012-2022 | 140 | 550 | 2107 | 2657 | 670 | Sandstone | 300 | Clay | Confined | Semi-confined | Semi-confined | Semi-confined | Semi-confined |
| *27* |  | *QX07* | 121°E 37.2°N | LN-3A | 1979 | 2012-2022 | 170 | 75.77 | 524.23 | 600 | 106 | Slate | 171 | Granite & slate | Confined | Confined | Semi-confined | Confined | Confined |
| *28* |  | *SH* | 117.3°E 37.2°N | LN-3A | 1982 | 2012-2022 | 178 | 2803 | 33 | 2836 | 37 | Limestone | 2799 | Shale | Confined | Confined | Semi-confined | Confined | Confined |
| *29* |  | *JN* | 117.7°E 35.4°N | LN-3A | 1972 | 2012-2022 | 146 | 20 | 221.44 | 320.2 | 68.1 | Limestone | 251.9 | Conglomerate & shale | Confined | Confined | Confined | Confined | Confined |
| *30* |  | *ZZ* | 117.4°E 34.9°N | LN-3A | 1979 | 2012-2022 | 110 | 239.63 | 261.98 | 501.61 | 261.98 | Sandstone | 239.63 | Mudstone | Confined | Semi-confined | Semi-confined | Confined | Semi-confined |
| *31* |  | *HZ* | 115.3°E 35.4°N | LN-3A | 1983 | 2007-2021 | 216 | 1138 | 863 | 2001 | 862 | Limestone | 1138 | Sandstone & mudstone | Confined | Confined | Semi-confined | Confined | Confined |
| *32* |  | *LC* | 116°E 36.4°N | DRSW-1 | 1998 | 2012-2022 | / | / | / | 2337.72 | 1552.72 | Limestone | 785 | Shale & mudstone | Confined | Confined | Confined | Confined | Confined |
| *33* | *Shanxi* | *JL* | 112°E 38.4°N | LN-3A | 1983 | 2007-2021 | 146 | 53.5 | 309.4 | 362.92 | 242.66 | Limestone | 120.26 | Limestone | Confined | Semi-confined | Semi-confined | Semi-confined | Semi-confined |
| *34* |  | *TY* | 112.4°E 37.7°N | LN-3A | 1968 | 2012-2022 | 220 | 482 | 283.78 | 765.78 | 285.78 | Limestone & dolostone | 480 | Limestone | Confined | Unconfined | Unconfined | Semi-confined | Unconfined |
| *35* |  | *SZDZT* | 112.5°E 39.3°N | LN-3A | 1987 | 2012-2022 | / | / | / | 694.75 | 301.72 | Limestone | 392.75 | Mudstone | Confined | Unconfined | Semi-confined | Confined | Semi-confined |
| *36* |  | *XY* | 111.8°E 37.2°N | LN-3A | 1982 | 2007-2021 | 150 | 502.47 | 99.27 | 502.93 | 102 | Sandstone & shale | 400 | Clay | Confined | Semi-confined | Confined | Confined | Confined |
| *37* |  | *LF* | 111.5°E 36.1°N | LN-3 | 1984 | 2012-2016 | 127 | / | / | 600.37 | 50 | Sand | 511 | Sand & clay | Confined | Semi-confined | Semi-confined | Semi-confined | Semi-confined |
| *38* |  | *QXDZT* | 112.2°E 37.4°N | LN-3A | 1979 | 2012-2022 | 146 | 291.65 | 150.54 | 442 | 150.35 | Shale | 291.65 | Clastic rock | Confined | Unconfined | Semi-confined | Confined | Semi-confined |
| *39* |  | *JX* | 111.9°E 37.2°N | LN-3A | 1983 | 2012-2022 | / | / | / | 315 | 221.15 | Sandstone & conglomerate | 93.85 | Clay & gravel | Confined | Confined | Semi-confined | Confined | Confined |

^#^Starting and ending periods for which data can currently be collected.

*Traditional method is based on qualitative determination using lithology. Some well information was not collected
